# Clustering cliques for graph-based summarization of the biomedical research literature

**DOI:** 10.1186/1471-2105-14-182

**Published:** 2013-06-07

**Authors:** Han Zhang, Marcelo Fiszman, Dongwook Shin, Bartlomiej Wilkowski, Thomas C Rindflesch

**Affiliations:** 1 Department of Medical Informatics, China Medical University, Shenyang, Liaoning 110001, China; 2 National Library of Medicine, Bethesda, MD 20894, USA; 3 DTUInformatics, Technical University of Denmark, Kongens Lyngby, Denmark; 4 Danish National Biobank, National Health Surveillance & Research, Statens Serum Institut, Copenhagen, Denmark

**Keywords:** Clique clustering, Graph-based summarization, Multi-document summarization, Semantic predications

## Abstract

**Background:**

Graph-based notions are increasingly used in biomedical data mining and knowledge discovery tasks. In this paper, we present a clique-clustering method to automatically summarize graphs of semantic predications produced from PubMed citations (titles and abstracts).

**Results:**

SemRep is used to extract semantic predications from the citations returned by a PubMed search. Cliques were identified from frequently occurring predications with highly connected arguments filtered by degree centrality. Themes contained in the summary were identified with a hierarchical clustering algorithm based on common arguments shared among cliques. The validity of the clusters in the summaries produced was compared to the Silhouette-generated baseline for cohesion, separation and overall validity. The theme labels were also compared to a reference standard produced with major MeSH headings.

**Conclusions:**

For 11 topics in the testing data set, the overall validity of clusters from the system summary was 10% better than the baseline (43% versus 33%). While compared to the reference standard from MeSH headings, the results for recall, precision and F-score were 0.64, 0.65, and 0.65 respectively.

## Introduction

Automatic summarization is emerging as a viable information processing mechanism to help users effectively access the large amount of textual data available online, especially in the biomedical domain. Such processing distils the most important information from source documents to produce an abridged condensate that serves as an informative and indicative summary of a given topic [1,2]. Summarization is often thought of as a natural language processing task due to the need for in-depth understanding of text to provide a useful summary. The analysis of source text may take various forms. In earlier work this was often limited to textual cues that identify salient information, while more recent research may involve concepts in a domain ontology [3] and semantic relation extraction [4,5].

**Figure 1:**
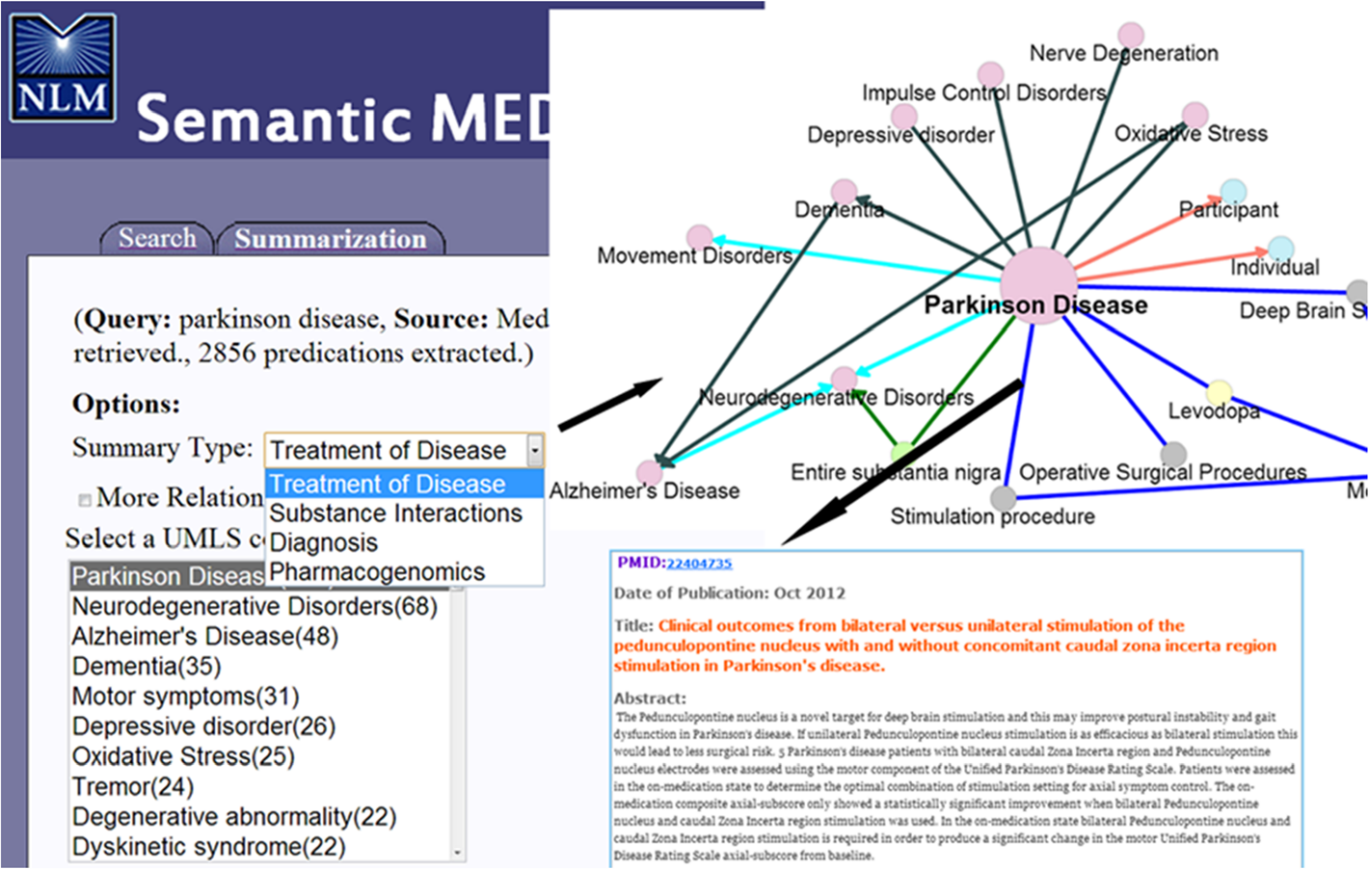
**Summary of 500 citations on Parkinson disease in Semantic MEDLINE.** This figure shows a snapshot of a graphical summary generated by Semantic MEDLINE. By selecting a schema and a summary theme, the user produces a graphical summary on a specific topic (Parkinson disease).

To facilitate access to the biomedical research literature, Fiszman et al. [6] developed an automatic abstractive summarization method based on semantic predications identified in biomedical text by the SemRep natural language processing system [5,7]. Both semantic predications and the abstractive summarization method are exploited in the Semantic MEDLINE Web application [8], which produces graphical summaries by condensing semantic predications found in MEDLINE citations (titles and abstracts) retrieved with PubMed queries. Relying on four manually predefined schemas, the user directs Semantic MEDLINE to generate summaries focused on several points of view, either treatment of disease, substance interactions, diagnosis, or pharmacogenomics. Nodes in the summary are concepts from the Unified Medical Language System (UMLS) [9] Metathesaurus, and arcs are semantic relations from the Semantic Network. By clicking on an arc, a user can obtain the sentence from which the relation was generated (Figure 1).

Although Semantic MEDLINE shows promise in managing the results of PubMed searches [10], it produces graphs that are too large and dense when generating summaries from more than a few hundred citations. This characteristic does not accommodate the thousands of citations that may be returned by a PubMed query (for example, nearly 150,000 for “breast cancer”). In earlier work [11], we exploited the graph theoretic notion of degree centrality to reduce large graphs by retaining only highly connected concepts. The method is effective in presenting readable, focused information to the user. However, it relies on predefined schemas, which must be devised for each topic point of view, and thus limit the applicability of this summarization methodology in covering the thematic diversity seen in the biomedical research literature.

In this paper, we explore a graph-based method to make automatic summarization more robust when confronted with large numbers of MEDLINE citations, without using predefined schemas. This multidocument method is based on a network representation of the semantic predications extracted from citations (titles and abstracts) returned by a PubMed query. Cliques are first identified in this graph and then clustered and labeled to identify several points of view represented in the summary. Since schemas are not used, the method is applicable to any biomedical topic.

The primary contribution of this paper is the application of graph theoretic constructs to semantic predications for automatic summarization in the biomedical domain. We also introduce a novel semantics-based criterion for determining final clusters, which is compared to a silhouette coefficient method. Finally, evaluations for cluster validity and accuracy, as well as the quality of the summary are also provided.

## Background

### SemRep semantic interpretation

The clustering method used for automatic summarization in this study depends on cliques identified in a graph of semantic predications extracted from PubMed citations with SemRep [5,7], a symbolic rule-based natural language processing system relying heavily on biomedical domain knowledge in the Unified Medical Language System (UMLS) [9]. Extraction of predications begins with an underspecified syntactic analysis based on the SPECIALIST Lexicon [12] and MedPost part-of-speech tagger [13]. MetaMap [14] maps simple noun phrases from this structure to Metathesaurus concepts, and “indicator rules” map syntactic elements to UMLS Semantic Network predicates. SemRep extracts 30 predicate types, in the domains of clinical medicine (e.g. TREATS, DIAGNOSES), substance interactions (e.g. INTERACTS_WITH, INHIBITS, STIMULATES), etiology (e.g. CAUSES, PREDISPOSES), and pharmacogenomics (e.g. AFFECTS, AUGMENTS, DISRUPTS)^.^Syntactic processing then identifies arguments (noun phrases mapped to Metathesaurus concepts) for each predicate. As an example, the predications (argument-predicate-argument) below were extracted from the text, *Patients with single brain lesion received an extra 3 Gy x 5 radiotherapy.*

Brain – LOCATION_OF – Single lesion

Single lesion – PROCESS_OF – Patients

Radiation therapy – ADMINISTERED_TO – Patients

### Automatic summarization

Automatic summarization condenses source text into an abbreviated version representing salient information. Most methods exploit an extractive process that selects informative text strings from the source and concatenates them into a summary. Fewer attempts have been made to generate an abstractive summary, which processes the source text and represents it using terms not found in the source. Both summarization techniques depend on identification of salient source content, either through informative textual cues, term frequency, or, more recently, graph-based metrics.

### Identifying salient source content

Most frequency-based methods provide extractive summaries composed of source sentences containing frequently occurring content units. Nenkova and Vanderwende [15] assessed the contribution of frequency of occurrence to summarization, which is considerable. Reeve et al. [16,17] further exploit domain ontologies to identify salient information.

Recently, graph structures have been used to represent source content to be summarized. Often, terms or sentences are represented as nodes and relations between them as arcs; however, abstractive representations are also used in graph-based analysis. Graph theory-based metrics have been proposed to identify salient information. Two commonly used metrics are degree centrality and eigenvector centrality, and both are based on connectedness. Degree centrality is determined by the connecting arcs a node has, normalized for the size of the graph, while eigenvector centrality is computed based on the connections a node has along with the connectedness of neighboring nodes. Several studies (e.g. [18-20]) have shown that degree centrality, when compared to other connectedness metrics, performs best for most tasks. LexRank [18] and TextRank [21] have applied connectedness metrics to generate multidocument summaries. In LexRank, for example, nodes represent sentences and arcs similarity between them. Node connectedness is used to identify prominent sentences as a summary.

In addition to text, biomedical data can also be represented as a graph, with nodes representing biological entities (e.g. genes or proteins) and edges associations between them. For example, protein-protein interactions can be successfully modeled by a graph. Based on the recognition of cohesive subgroups (such as cliques), gene or protein complexes can be extracted to help predict protein interactions or find gene-disease relations [22,23].

### Cliques in graph theory

**Figure 2:**
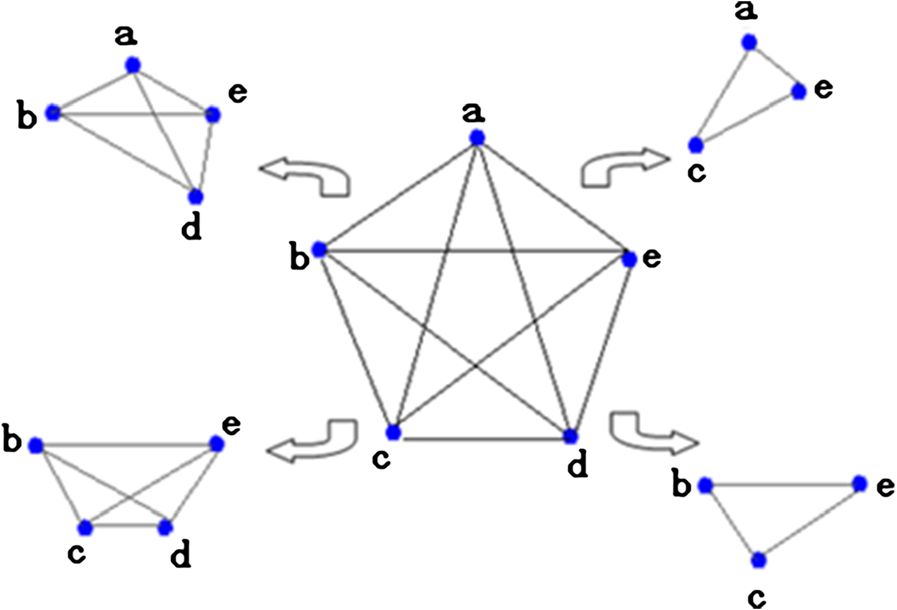
**Cliques.** The clique in the center is the maximal clique. The four smaller cliques contained in it are displayed on the periphery.

In graph theory, a clique is a subgraph in which every node is connected to every other node in that subgraph. The size of a clique is defined by the number of nodes in it. For example, a 4-clique contains four nodes. Figure 2 shows five cliques, one 5-clique at the center, two 4-cliques on the left, and two 3-cliques on the right. In Figure 2, the four peripheral smaller cliques are included in the 5-clique, which is a maximal clique not included in any other.

Identifying cliques can help find cohesive subgroups in a graphical network. Usually, each node in a clique is, in some way, highly related to every other node. This characteristic makes clique identification a very important approach to uncover meaningful groups from a network, such as protein-complex discovery from protein-protein interaction networks [24], collaborating groups from co-authorship networks [25], etc. Zubcsek et al. [26] clustered cliques to identify information communities with UCINET. Taking advantage of node overlap among cliques, Ah-Pine and colleagues [27] proposed a clique-based clustering method to annotate named entities.

### Theme identification

Identification of various themes contained in the summary can help users locate specific information they are interested in and link to relevant source documents. Theme identification, also known as topic identification or topic discovery, is the process of assigning one or more labels to text [28]. To discriminate it from the topic of a summary, we refer to this task as “theme identification” in this paper.

Theme identification is particularly important in multi-document summarization. To avoid similar information repetitiously appearing in the summary, Stein et al. [29] grouped their summaries from single documents into clusters and selected a representative passage from each cluster to construct the final summary. Other studies [28] clustered documents before performing summarization in order to help users select clusters of interest.

Clustering is a very powerful data mining technique for identifying and labeling themes in a group of documents, and both k-means and hierarchical clustering are used for this task. For each cluster, features, such as keywords, terms, or sentence are chosen as the label (or theme). K-means clustering [30,31] groups documents into predefined n classes. It is often used when the number of classes for the documents is known and serves as a reference standard to evaluate the final clusters generated. In reality, it may be hard to obtain expert-determined classification for thousands of biomedical documents, and hierarchical clustering is often used instead.

Hierarchical clustering is an unsupervised method that does not require setting a predefined number of clusters for the documents. When using hierarchical clustering to group documents and generate labels for the clusters, the vector space model is often adopted to produce term or keyword vectors, which help indicate similarity among documents [30,32,33]. Subsequently, documents are clustered into several subgroups, and terms or keywords that are salient for a given cluster are extracted as the theme (or label) for the cluster.

## Methods

### Overview

**Figure 3:**
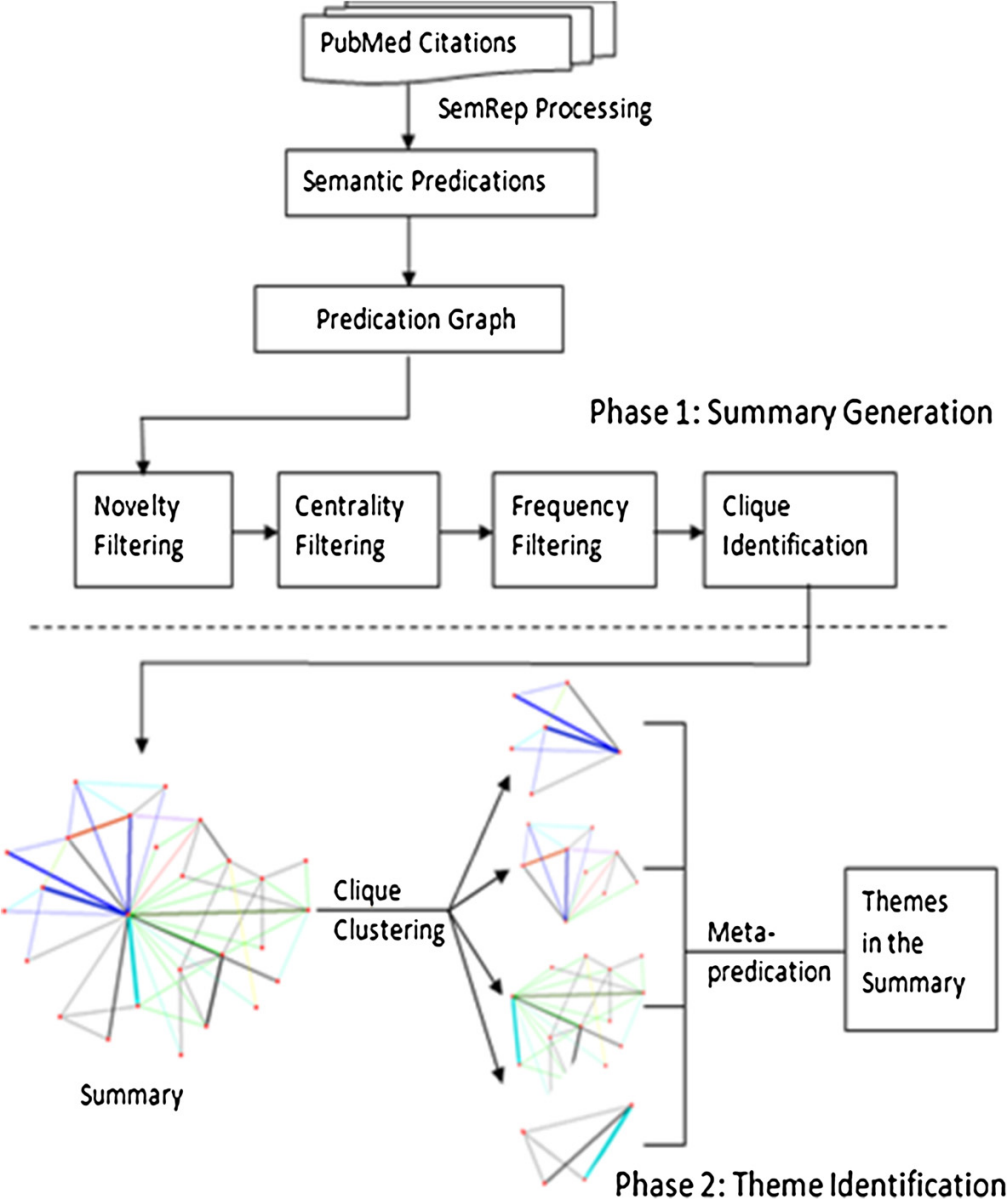
**Work flow of the summarization processing.** The system includes two phases in processing. In the first phase, semantic predications extracted from MEDLINE citations with SemRep are represented in a graph. Then four processing steps (novelty filtering to eliminate uninformative predications, node centrality filtering, frequency filtering, and clique identification) successively condense the predication graph into a graphic summary. In phase two, clique clustering, an application of the UCINET, is used to partition the summary into several clusters representing summary themes. The theme of the clusters is labeled with a metapredication.

Our method for automatic summarization includes two major parts: (1) applying graphical metrics to help produce a summary and (2) using a clustering technique as well as semantics to identify themes contained in the summary (work flow shown in Figure 3). In the first part, the citations from a PubMed search are represented as semantic predications with SemRep. Then predications are converted into a graph with arguments as nodes and predicates as arcs. Degree centrality and frequency of occurrence of predications are used to eliminate nonsalient predications from the graph, thus identifying relationships important for the summary. Finally, cliques are identified in the summarized graph. In the second part, theme identification, a hierarchical clustering algorithm is applied to cliques to group them into several clusters, and a semantic theme label is assigned to each cluster.

### Data sets

To test the effectiveness of our method for automatic summarization, several topics, including disorders, substances, and physiological processes, were chosen for training and testing. Citations were retrieved from MEDLINE with the topic term or phrase as major MeSH term, limited to English and with abstracts. To accommodate evaluation, we further limited the number of citations to fewer than 20,000, by publication date (although the system can process any number of citations). The topics and the number of the citations for training and testing are shown in Table 1.

**Table 1:** Topics for training and testing sets

**Training**		**Testing**	
**Query terms**	**No. of citations**	**Query terms**	**No. of citations**
Heart Diseases/therapy	15,301	Diabetes Mellitus	14,947
Migraine Disorders	4250	Lung Neoplasms/genetics	4826
Parkinson Disease	10,497	Schizophrenia	16,799
Digestion	3808	Hypersensitivity	9908
Inflammation	9300	Oxidative stress	18,007
Sleep	17,659	Hydrocortisone	12,948
Anti-HIV Agents	8535	Anti-inflammatory Agents, Non-steroidal	15,365
C-Reactive Protein	6085	Heat-Shock Proteins	11,502
Flavonoids	11,381	Interleukin-6	12,959
Genes, p53	6512	Hydroxymethylglutaryl-CoA Reductase Inhibitors	6837
Nitric Oxide	17,577	Tumor Necrosis Factors	11,021

### Graphical representation for semantic predications

**Figure 4:**
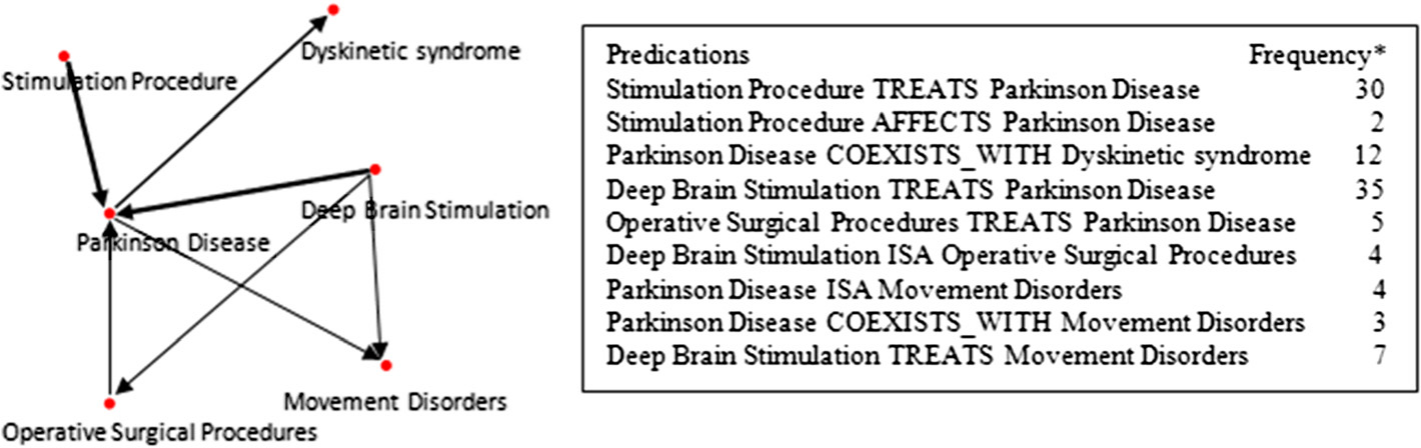
**An example of a predication graph.** The graph on the left was produced from the predications listed on the right, with frequency of occurrence given next to them. Predications with the same arguments but different predicates may occur in a graph, such as “Parkinson Disease ISA Movement Disorders” and “Parkinson Disease COEXISTS_WITH Movement Disorders”. Such predications are represented as a single arc with multiple labels joining the argument nodes. For clarity, arc labels are not shown in Figure 4.

SemRep predications extracted from MEDLINE citations to be summarized are first converted to a graph, with nodes representing arguments and arcs predicates. The direction of the arcs is from subject to object. The width of the arcs indicates the number of citations containing a given predications in the entire input set. Figure 4 shows the graph resulting from processing a sample set of 9 distinct predications (on the right).

### Summarization processing

Besides frequency of occurrence of predications, we used two graphical constructs, degree centrality and cliques, to condense the graph into a summary of salient predications. Both degree centrality and clique detection help identify predications with high connectedness, which, along with frequently occurring arcs in the graph, convey information crucial to the summary. For example, in Figure 4, the predication “Deep Brain Stimulation TREATS Parkinson Disease” is identified as highly salient because the nodes representing its arguments have more connections than other nodes, and the frequency of occurrence of the arc between them is higher than that of other arcs.

### Eliminate uninformative predications

Before graph theoretic techniques are applied to create a summary, predications with at least one generic argument are eliminated from the graph, which removes uninformative relationships as part of the condensing process of summarization [1,2]. As noted earlier, arguments are Metathesaurus concepts, and generic arguments, (e.g. “Patients”) are identified as occurring higher than an empirically determined cutoff in the UMLS hierarchy [6]. For example, the predication “Pharmaceutical Preparations TREATS Parkinson Disease” is eliminated from the graph, while “Dopamine Agonists TREATS Parkinson Disease” is kept because “Pharmaceutical Preparations” in the former is high in the hierarchy, while both arguments in the second predication are lower.

### Identify highly connected nodes

We assume that central nodes in the predication network are likely to represent important contents in the documents being summarized. In our previous study [11], we found that degree centrality effectively identifies information crucial to summarization for researchers and clinicians. In the current study, we used degree centrality to sort the concepts in the network, and then, based on training data, defined a degree centrality cutoff, which is the mean of the sum of the degree centrality scores plus half of the standard deviation. Predications in which both arguments have a degree centrality score above the cutoff are kept, while others are eliminated.

### Eliminate predications with lower frequency of occurrence

Since frequency also plays an important role in automatic summarization, we calculated frequency of occurrence for the rest of the predications. The computation for frequency is based on how many citations a predication appears in [34]. (When a predication occurs more than once in a single sentence, we count that occurrence as one.) A formula similar to that for degree centrality (the mean of the sum of the frequency of occurrence, plus half of the standard deviation) was adopted and predications with frequency of occurrence below the cutoff were eliminated from the graph.

### Identify cliques

After the first three steps, the predications remaining were those with high frequency of occurrence and having highly connected arguments; in the next step, cliques were identified in the graph of these predications. The tool used to identify cliques and cluster them in the next step is UCINET 6 [35], a social network analysis package particularly useful for extracting cliques and analyzing overlap [36]. There is other research of relevance to our work. Boyack et al. [37] compare the effectiveness of several algorithms for clustering large numbers of documents, but they do not address details of the semantic content involved. Blondel et al. [38] discuss an efficient algorithm for identifying communities in large networks, but the “content” of these involves only one feature, primary language used in mobile phone networks, rather than the rich expressiveness of SemRep semantic predications. Since our main concern is exploiting semantic predications for the semantic content of documents, for the purpose of automatic summarization, rather than development of clustering algorithms, UCINET is entirely adequate.

The UCINET algorithm to identify cliques is based on the notion of a maximal clique, one that is not contained in any other. Cliques are allowed to overlap, which means that concepts can be members of more than one clique. This feature is important for summarization because it permits certain concepts, which have high degree centrality and are the core of a network (such as the topic of the summary) to appear in several cliques of the graph.

### Theme identification

#### Overview

A summary of a large number of documents usually includes several themes, or points of view. For example, a summary of breast cancer may include information on chemotherapies, procedures, genetic etiology, etc. In exploiting such a summary, a user may want to focus on any one of these themes. The accessibility of a summary is increased if the different themes are discriminated from each other and overtly represented. Although cliques correlate somewhat with themes, this is not absolute due to the fact that cliques share nodes.

Our approach to identifying themes in a summary exploits clusters of cliques and has two phases. In the first, clustering is based solely on nodes in the clique (which represent arguments in the semantic predications constituting the cliques). In addition to identifying cliques from the predication network, UCINET automatically produces a clique co-membership matrix and a hierarchical clique clustering, which produces several possible solutions, each containing a varying number of clusters.

We then use semantic processing to select the clustering solution that best represents the themes of the summary. The goal is to put cliques with similar themes in the same cluster, while keeping cliques with different themes in separate clusters. The challenge is to determine the best clustering solution by grouping cliques in such a way that the themes of the summary are optimally represented. Generally, the best clustering solution is neither too compact (a single cluster containing all cliques) nor too dispersed, as is the case if every cluster is a singleton having only one clique. When this solution has been selected, further processing determines whether some of the clusters should be collapsed [39] based on semantic similarity.

#### Visualizing cluster solutions

The tool we used to find and cluster cliques is UCINET [35], a hierarchical clustering software package originally developed for social network analysis. UCINET produces a clique co-membership matrix in which the (i,j)th entry of the matrix is the number of shared nodes (arguments) in clique i and clique j and the diagonal entries are the size of the cliques. Based on this matrix, UCINET produces an icicle plot composed of solutions to the clique clustering. Although each clique is assigned to a unique cluster, concepts may be in more than one cluster [40].

**Figure 5:**
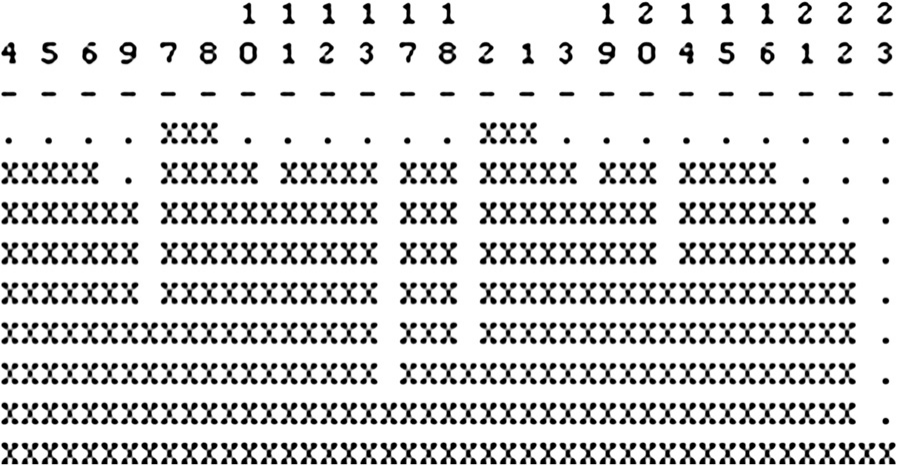
**An icicle plot of clustered cliques for Parkinson disease.** An icicle plot represents the clustered cliques for Parkinson disease produced by UCINET. The summary for Parkinson Disease contains 23 cliques, whose numeric labels are displayed on the top of the icicle plot. There are 9 cluster solutions proposed for Parkinson Disease; each cluster solution is represented with a single row below the clique labels.

These solutions can be visualized as an icicle plot [41] such as that seen in Figure 5, in which the numbers on the top of the plot are labels for each clique. Each row shows a cluster solution with a different number of clusters. For example, in the bottom row all cliques are included in one cluster, while in the top row there are two multiclique clusters: the first contains cliques 7 and 8, while the second contains cliques 2 and 1. All other clusters in the top row are singletons containing a single clique. We use an icicle plot to guide the processing for selecting the clustering solution that best represents the themes of a summary our system produces.

#### Semantic processing for labeling clusters

In our method, determining the best clustering solution is based on semantic similarity of the individual clusters, as represented by theme labels. Our graphs represent semantic predications, and cliques thus contain the arguments of predications (as nodes) along with the predicates connecting them. Identifying similarity of clusters depends on characteristics of the predications contained in the cliques that constitute the clusters. Other approaches have used terms or sentences as theme labels, neither of which provides the greater expressiveness of semantic predications.

Metapredications are used to identify and label the theme for each cluster. A metapredication, whose form is similar to a SemRep predication, is defined as “<Semantic Group > <Predicate Group > <Semantic Group>”. The scope of the semantic group and predicate group is broader than that of the arguments and predicate of a SemRep predication, so a metapredication generalizes the meaning of a cluster of cliques composed of several predications.

The predicate group used in a metapredication was defined for this project as a group of SemRep predicates expressing related content. For example, predicate PART_OF is used with two physical units, in which the first is a component (or division) of the second. Predicate LOCATION_OF is used to indicate the body location (or region) of an entity or the site of a process. Since these two predicates both express a relationship between physical entities, they were put into the predicate group Physical. The SemRep predicates that were assigned to predicate groups are given in Table 2.

**Table 2:** Predicate groups

**Predicate group**	**Predicates**
Physical	part_of, location_of
Interaction	inhibits, stimulates, interacts_with
Therapy	treats, prevents, compared_with*, uses**
Causation	associated_with, causes, predisposes
Diagnosis	diagnoses, measures
Affects	affects, augments, disrupts
Comorbidity	coexists_with

*COMPARED_WITH regularly appears along with TREATS to indicate drug comparisons.

** USES is commonly seen with TREATS to specify some aspect of the therapy.

The arguments of a metapredication are groups of semantic types suggested by McCray et al., who aggregated 134 UMLS semantic types into 15 groups based on six principles (semantic validity, parsimony, completeness, exclusivity, naturalness and utility) [42]. For example, the semantic type ‘Therapeutic or Preventive Procedure’ and ‘Laboratory Procedure’ belong to the semantic group Procedures, while ‘Disease or Syndrome’ and ‘Neoplastic Process’ are included in Disorders. The metapredications used for this project are given in Table 3 and represent the major SemRep semantic predications extracted from MEDLINE titles and abstracts.

**Table 3:** Metapredications

**Metapredication**	**<Semantic group > <Predicate group > <Semantic group>**
Body location	<Anatomy > <Physical > <Anatomy>
	<Anatomy > <Physical > <Chemicals & Drugs>
	<Anatomy > <Physical > <Disorders>
Substance interaction	<Chemicals & Drugs > <Interaction > <Chemicals & Drugs>
	<Chemicals & Drugs > <Interaction > <Genes & Molecular Sequences>
Drug treatment	<Chemicals & Drugs > <Therapy > <Disorders>
	<Chemicals & Drugs > <Therapy > <Chemicals & Drugs > *
Procedure treatment	<Procedures > <Therapy > <Disorders>
	<Procedures > <Therapy > <Chemicals & Drugs > **
Etiology	<Genes & Molecular Sequences > <Causation > <Disorders>
	<Chemicals & Drugs > <Causation > <Disorders>
	<Disorders > <Causation > <Disorders>
Diagnosis	<Procedure > <Diagnosis > <Disorder>
	<Procedure > <Diagnosis > <Chemicals & Drugs > ***
Affect	<Disorder > <Affects > <Disorder>
	<Chemicals & Drugs > <Affects > <Disorder>
	<Chemicals & Drugs > <Affects > <Physiology>
Disease comorbidities	<Disorders > <Comorbidity > Disorders

*The predicate for this Metapredication is COMPARED_WITH.

** The predicate for this Metapredication is USES.

*** The predicate for this Metapredication is MEASURES.

In theme identification, each SemRep predication in a cluster identified in the icicle plot is assigned to a metapredication. For example, the predications “Dopamine Agonists TREATS Parkinson Disease” and “Dopamine Agonists TREATS Dyskinetic syndrome” are assigned to the metapredication “<Chemicals & Drugs > <Therapy > <Disorders>” because the predicate TREATS belongs to the predicate group < Therapy > and the semantic type of the subjects and the objects of these two predications belongs to the semantic group Chemicals & Drugs and Disorders, respectively. Metapredications are then counted and sorted in descending order of frequency of occurrence; the most frequent identifies the theme of the cluster and serves as its label.

**Figure 6:**
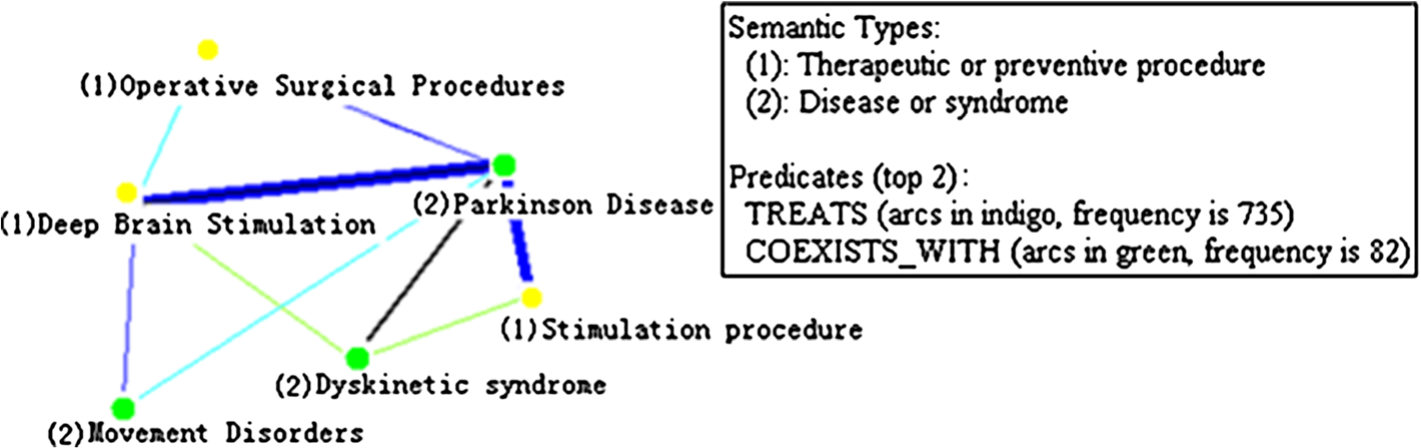
**Theme label for of cluster 1 in Figure**5**: Procedure treatment (<Procedures > <Therapy > <Disorders>).** The number enclosed to the left of each node concept in the graph indicates the semantic type of that concept, as shown in the box on the right. The two most frequently occurring predicates are also given in the box. The visualization was performed using Pajek.

As an example of assigning a metapredication theme label to a cluster of cliques, the graphical representation of the first cluster in row 3 in Figure 5 (clique 4-5-6-9) is shown on the left side of Figure 6. For this cluster, there are two semantic types, ‘Therapeutic or Preventive Procedure’ and ‘Disease or Syndrome’, which belong to two semantic groups, Procedures and Disorders respectively. Given that the most frequent predicate in this cluster is TREATS, the most frequent metapredication is “Procedure treatment” (<Procedures > <Therapy > <Disorders>), which serves as the theme label for this cluster.

#### Selecting the optimal clustering solution

Semantic theme labels form the basis for selecting the best clustering solution to represent themes for the summary generated by the method. As represented in the icicle plot, the several clustering solutions are arranged hierarchically, so that the solution containing the most clusters is at the top of the plot. In each succeeding row, adjacent clusters may be merged (based on shared nodes in the cliques being clustered), so that the final, bottom row contains fewer clusters than those preceding it. In our method, merging of clusters in succeeding rows is augmented with semantic processing to choose the optimal clustering solution, one in which there are no clusters that could be merged in the succeeding solution (row) based on shared nodes and which have the same theme label.

After theme labels have been computed for clusters in the icicle plot, each successive row of the icicle plot is processed, starting with the row that is likely to require minimum merging. Based on training data, this is the first row from the top that has no more than three singleton clusters (containing only one clique). The current row is compared to the immediately succeeding row, and it is noted whether any separate clusters in the current row are merged in the following row, and further, whether those clusters have the same metapredication theme label. If both conditions are satisfied, the succeeding row is considered to be a better solution than the current row, and the former succeeding row becomes the current row. When a row is encountered for which the succeeding row is not a better solution than the current row, the current row is considered the optimal solution.

**Figure 7:**
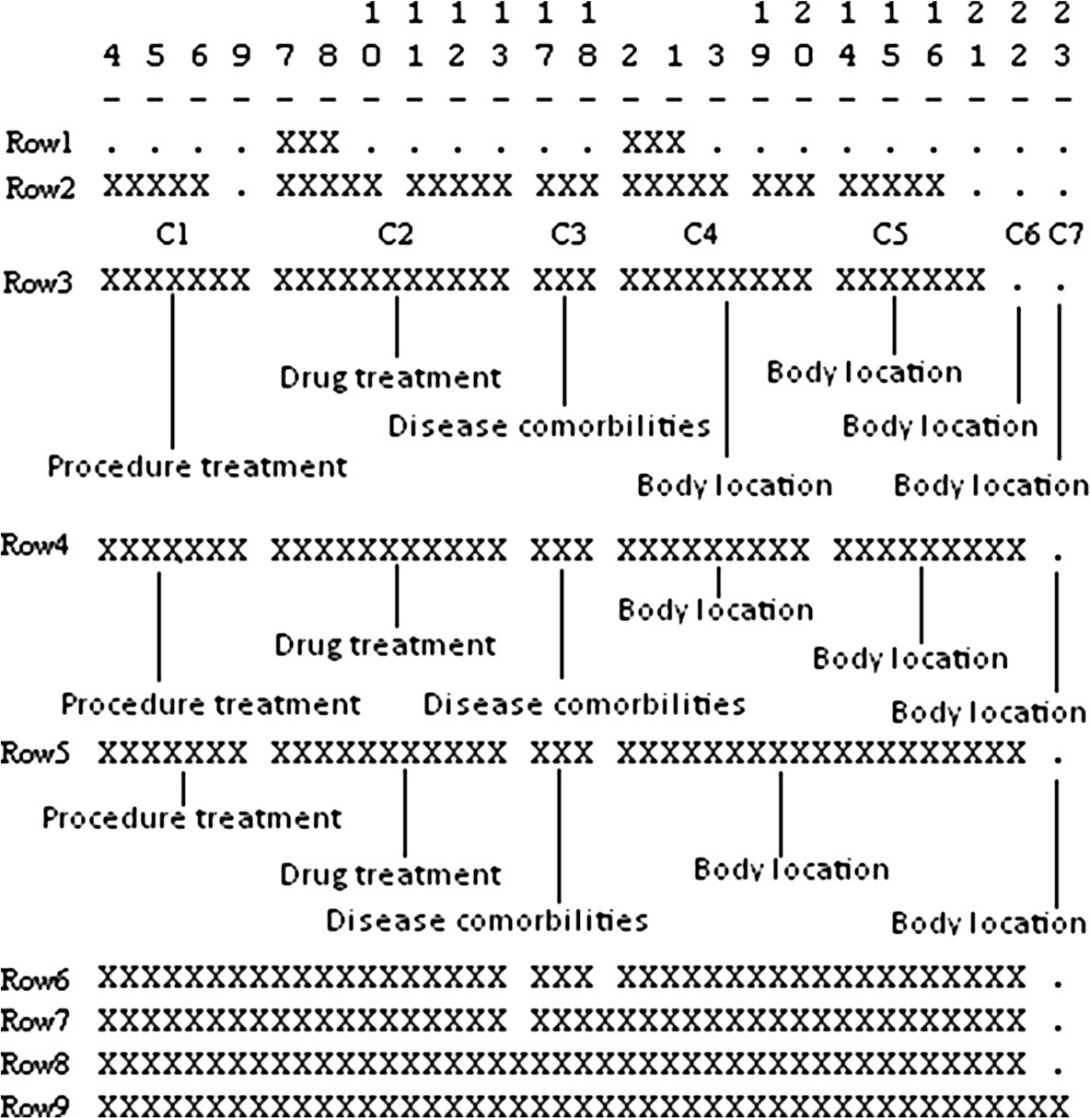
**Figure**5**labeled with summary themes.** Figure 7 illustrates how the optimal cluster solution is selected by labeling the clusters contained in 3 solutions (row 3 to row 5). The cluster labels are displayed below the three solutions. The cluster merging process starts at row 3. In the succeeding line, clusters with the same labels are merged together.

For example, in Figure 7, row 3 is the starting line because it has only two singleton clusters. In the succeeding line, row 4, clusters 5 and 6 (C5 and C6) are merged, and they have the same theme label (Body Location). Thus row 4 is considered a better solution than row 3. When row 4 is compared to row 5, it is seen that clusters 4 and 5–6 have the same theme label (Body Location) and that they are merged in row 5, which is thus considered to be a better solution than row 4. When the immediately succeeding row, row 6, is encountered, it is seen that clusters 1 and 2 are merged. Since they do not have the same label theme, the algorithm stops and row 5 is selected as the best clustering solution for this summary.

#### Evaluation

In a previous study [11], we evaluated the effectiveness of degree centrality as a condensing mechanism for automatic summarization to answer disease treatment questions in a semantic predication graph. We have also constructed a semantic predication gold standard [43] to support further evaluation. In addition, we have assessed the ability of Semantic MEDLINE, a SemRep-based summarizer, to identify useful drug interventions for evidence-based medical treatment [10]. In this paper, we evaluated two aspects of clustering cliques for automatic summarization: the validity of the clusters produced and the quality of the cluster labeling.

#### Validity of the clusters

The validity of the clusters was assessed by measuring cluster cohesion and cluster separation. Cohesion measures the purity of the objects within a cluster, i.e. how closely related the objects in a cluster are. Separation measures the isolation of the objects in different clusters, i.e. how distinct a cluster is from other clusters. For our clusters, composed of semantic predications, we evaluated how related the semantic predications in a cluster are to its cluster label (cohesion) and how well-separated semantic predications with different labels are in different clusters.

For example, for a cluster *i* labeled as “Procedure treatment” (Procedures < Therapy > Disorders), if there are *x* predications included in this metapredication (such as “Deep Brain Stimulation TREATS Parkinson Disease”) and *y* predications not matching (such as “Dyskinetic syndrome COEXISTS_WITH Parkinson Disease”), then the cohesion of the cluster *i* is defined as:

$$\mathrm{Coh}\left(C_{i}\right)=x/\left(x+y\right)$$

If in addition to cluster *i*, there are *z* predications in other clusters that match the label of cluster *i*, then the separation of cluster *i* is:

$$\mathrm{Sep}\left(C_{i}\right)=x/\left(x+z\right)$$

Inspired by calculation of F-score for information retrieval task, we defined the overall validity of cluster *i* as follows:

$$\mathrm{Overall}\mathrm{validity}\;\left(C_{i}\right)=2\mathrm{Coh}\left(C_{i}\right)\mathrm{Sep}\left(C_{i}\right)/\left(\mathrm{Coh}\left(C_{i}\right)+\mathrm{Sep}\left(C_{i}\right)\right)$$

We compared our system output to a baseline whose clique clusters were determined by the silhouette coefficient [44], which is often used to determine the appropriate number of clusters in clustering data mining research. We used a symmetric matrix in which each cell was the number of shared nodes by the corresponding pair of cliques to compute the distance between cliques. Then the average silhouette coefficient (ASC) (see [45] for details) was calculated for each clustering solution and the solution with the highest ASC served as the baseline. Cohesion, separation, and overall validity were also calculated for the baseline.

#### Quality of the cluster labeling

The accuracy of themes annotated by cluster labels is important to the final summary. A cluster with a poor label may be ignored by users even if it links to a group of documents relevant to their information needs. We thus also evaluated the labeling effectiveness of our system. Since it is almost impossible for domain experts to produce class labels for the results of clustering tens of thousands articles, we constructed a reference standard for evaluation based on the medical subject heading (MeSH) descriptors assigned to source citations that produce predications in the summary clusters. This evaluation was done by comparing arguments extracted from the predications in the cluster to MeSH indexing terms assigned to the citations from which the predications were extracted.

For each citation in MEDLINE, indexers at the National Library of Medicine assign 5 to 15 MeSH descriptors as well as qualifiers (if necessary) to cover the topics of the article; they also indicate those MeSH descriptors reflecting the major points of the article as major MeSH descriptors. Since this indexing procedure is performed by human experts, it is deemed that the MeSH descriptors, especially the major ones, accurately represent the contents of the article. For example, for a citation entitled “Aspirin and antiplatelet agent resistance: implications for prevention of secondary stroke” (PMID: 20932071), the major MeSH descriptors are: Aspirin/pharmacology; Platelet Aggregation Inhibitors/pharmacology; Stroke/prevention & control. In constructing the reference standard, we ignored MeSH qualifiers. For example, MeSH descriptors “Antipsychotic Agents/therapeutic use” and “Antipsychotic Agents/administration & dosage” were counted as one term.

For each cluster in the summary, major MeSH descriptors assigned to citations producing predications in the given cluster were extracted and sorted in descending order of frequency of occurrence. The predication arguments in each cluster were compared to an equal number of the ranked MeSH descriptors, starting with the most frequent descriptor.

In comparing predication arguments to MeSH indexing terms, we exploited Metathesaurus synonymy to match concepts in the graph to MeSH descriptors. For example, the concept “Diabetes Mellitus, Non-Insulin-Dependent” was matched to term “Diabetes Mellitus, Type 2” because the concept is a synonym of the term in MeSH vocabulary. Finally, recall, precision and F-score were calculated.

## Results

### An example of the final summary

**Figure 8:**
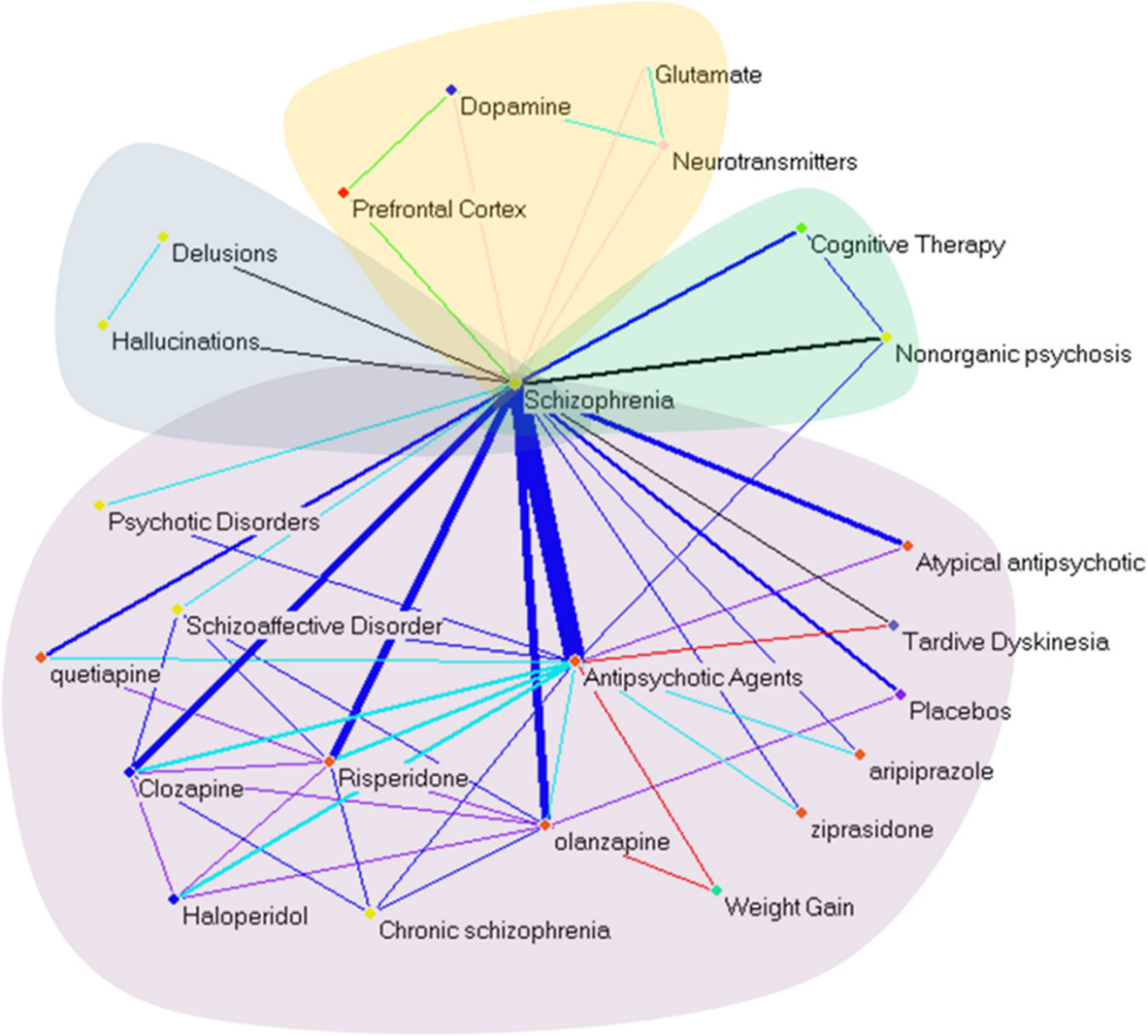
**Summary and theme partitions for schizophrenia.** To highlight the clusters within the summary, clusters with different themes are manually marked in different background colors: Etiology (yellow), Procedure treatment (green), Drug treatment (violet), and Disease comorbidities (gray).

Figure 8 illustrates the final graphical summary (produced with Pajek [46]) for the topic schizophrenia, which appears as the central node of the graph. Four themes were identified and are highlighted in color. Notably in this summary, delusions and hallucinations are seen as comorbidities of schizophrenia, while dopamine, glutamate and neurotransmitters are associated with its etiology. Drug treatment constitutes the largest cluster; in addition to representing major drugs for schizophrenia (linked by blue TREATS arcs), it shows comparison between two drugs (purple arcs, COMPARED_WITH), and some adverse effects resulting from the drugs, such as weight gain and tardive dyskinesia (red arcs, CAUSES). It should be noted that the characteristics of this graph reflect the condensing aspects of a summary, and do not necessarily accommodate other information management tasks, such as discovery. For an example of processing semantic predications for discovery see [47].

### Validity of the clusters

Table 4 shows cohesion, separation and overall validity of both the system summary (SS) clusters and the baseline (BL, Silhouette clusters) for the testing data. Out of 11 topics, one, interleukin-6, produced only one cluster, so we did not compute cohesion and separation for this topic. Results for the other 10 topics appear in Table 4.

**Table 4:** Validity of the clusters for system summary (SS) and baseline (BL)

**Topic**	**Cohesion**		**Separation**		**Overall Validity**	
	**SS**	**BL**	**SS**	**BL**	**SS**	**BL**
Diabetes Mellitus	0.38	0.41	0.70	0.22	0.46	0.29
(95% CI)	(0.32-0.45)	(0.35-0.48)	(0.64-0.76)	(0.17-0.28)	(0.43-0.56)	(0.23-0.35)
Lung Neoplasms	0.46	0.51	0.51	0.22	0.48	0.31
(95% CI)	(0.34-0.57)	(0.40-0.62)	(0.39-0.63)	(0.13-0.31)	(0.36-0.60)	(0.20-0.40)
Schizophrenia	0.81	0.86	0.91	0.35	0.86	0.50
(95% CI)	(0.70-0.92)	(0.76-0.96)	(0.84-0.99)	(0.22-0.48)	(0.76-0.96)	(0.36-0.64)
Hypersensitivity	0.46	0.54	0.38	0.23	0.42	0.32
(95% CI)	(0.37-0.54)	(0.46-0.63)	(0.30-0.46)	(0.16-0.30)	(0.33-0.50)	(0.24-0.40)
Oxidative stress	0.39	0.52	0.35	0.18	0.37	0.27
(95% CI)	(0.32-0.46)	(0.45-0.58)	(0.28-0.41)	(0.13-0.23)	(0.30-0.43)	(0.21-0.32)
Hydrocortisone	0.54	0.54	0.59	0.59	0.56	0.56
(95% CI)	(0.47-0.62)	(0.50-0.58)	(0.51-0.66)	(0.55-0.63)	(0.49-0.64)	(0.52-0.60)
Heat-Shock Proteins	0.54	0.54	0.24	0.16	0.33	0.25
(95% CI)	(0.47-0.60)	(0.47-0.60)	(0.19-0.30)	(0.11-0.20)	(0.27-0.40)	(0.19-0.29)
Anti-inflammatory Agents, Non-steroidal	0.49	0.48	0.71	0.43	0.58	0.45
(95% CI)	(0.42-0.56)	(0.41-0.55)	(0.64-0.77)	(0.37-0.50)	(0.51-0.65)	(0.38-0.52)
Hydroxymethylglutaryl-CoA Reductase Inhibitors	0.38	0.43	0.35	0.24	0.36	0.31
(95% CI)	(0.31-0.45)	(0.36-0.50)	(0.28-0.42)	(0.18-0.29)	(0.29-0.43)	(0.24-0.37)
Tumor Necrosis Factors	0.53	0.51	0.31	0.54	0.39	0.52
(95% CI)	(0.45-0.61)	(0.42-0.60)	(0.23-0.38)	(0.46-0.63)	(0.31-0.47)	(0.44-0.61)
Overall	0.47	0.51	0.40	0.24	0.43	0.33
(95% CI)	(0.45-0.50)	(0.49-0.54)	(0.38-0.42)	(0.22-0.26)	(0.41-0.46)	(0.31-0.35)

### Quality of the summary theme labeling

Table 5 shows the results of comparing predications in the summary to MeSH terms. The overall F-score is 0.65, with recall 0.64 and precision 0.65. Scores are largely consistent across all eleven topics and are comparable to those obtained with other systems (e.g. [27]).

**Table 5:** System output compared to MeSH indexing

**Topic**	**Recall**	**Precision**	**F-score**
Diabetes Mellitus	0.61	0.62	0.61
(95% CI)	(0.50-0.71)	(0.51-0.72)	(0.52-0.70)
Lung Neoplasms	0.71	0.79	0.75
(95% CI)	(0.57-0.85)	(0.66-0.93)	(0.62-0.88)
Schizophrenia	0.86	0.89	0.87
(95% CI)	(0.77-1)	0.73-0.89)	0.76-0.99)
Hypersensitivity	0.75	0.73	0.74
(95% CI)	(0.65-0.86)	0.63-0.84)	0.65-0.83)
Oxidative stress	0.58	0.62	0.60
(95% CI)	(0.47-0.68)	(0.51-0.73)	(0.51-0.69)
Hydrocortisone	0.77	0.77	0.77
(95% CI)	(0.67-0.87)	(0.67-0.87)	(0.68-0.86)
Heat-Shock Proteins	0.49	0.52	0.50
(95% CI)	(0.39-0.60)	(0.41-0.62)	(0.42-0.59)
Anti-inflammatory Agents, Non-steroidal	0.60	0.57	0.58
(95% CI)	(0.47-0.72)	(0.45-0.69)	(0.48-0.68)
Hydroxymethylglutaryl-CoA Reductase Inhibitors	0.78	0.75	0.77
(95% CI)	(0.67-0.89)	(0.65-0.86)	(0.67-0.86)
Tumor Necrosis Factors	0.64	0.73	0.68
(95% CI)	(0.53-0.75)	(0.62-0.83)	(0.59-0.78)
Interleukin-6	0.49	0.47	0.48
(95% CI)	(0.37-0.62)	(0.35-0.59)	(0.38-0.58)
Overall	0.64	0.65	0.65
(95% CI)	(0.61-0.68)	(0.62-0.69)	(0.62-0.68)

## Discussion

Generally, results showed that our method, based on graph theory as well as semantic predications, can produce satisfying summaries of large numbers of biomedical documents. The validity of clusters determined by semantics was better than that determined by the Silhouette Coefficient, and, further, the summary represented the major salient content of topics. Analysis of the overall validity of clusters showed that system output is 10% better than the baseline (43% versus 33%). Although the cohesion of the baseline is slightly higher than that of the system summary, the separation of the system summary is significantly better than that of the baseline. The number of clusters determined by the Silhouette Coefficient is greater than the number determined by semantic information, which results in a relatively higher cohesion and lower separation in the baseline.

We used metapredications to calculate cohesion and separation; such predications were constructed from semantic information pertinent to the core meaning of the themes. For example, the drug therapy theme (<Chemicals & Drugs > <Therapy > <Disorders>) expresses predications asserting specific drug therapies (TREATS) and comparison of such therapies (COMPARED_WITH).

Predications that do not belong to these two metapredications are counted as false positives when computing cohesion and separation. A problem arose with the predicate CAUSES, which SemRep uses to expresses both side effect of drug (which would be reasonable to include in the drug therapy theme) and etiology of disease (which is not in the scope of this theme). We chose not to include CAUSES in this theme, which caused some legitimate side-effect predications to be considered false positives when evaluating this theme. This decreased cohesion and separation, as well as overall validity for clusters containing the drug therapy theme.

### False negatives

Two issues were encountered in comparing concepts in each cluster to MeSH descriptors to evaluate the summary, both of which caused discrepancy between results and actual quality of the summary in expressing the semantic content of input citations. The first issue is due to indexing policy. For example, concepts referring to body part represented the major contents in disease location clusters. However, MeSH descriptors in the anatomy category are not normally indexed as major topics. For example, lung (location of lung cancer) and pancreas (location of insulin), were not indexed as major topics.

A second problem encountered in matching predications to MeSH indexing terms involves qualifiers (subheadings). For example, the concept “Toxic effects” in the predication “Anti-inflammatory Agents, Non-steroidal CAUSES Toxic effects” was often extracted from citations that had been indexed with the qualifier “toxicity.” Since only MeSH descriptors were compared in the evaluation, this concept was counted as a false negative.

### False positives

False positives were largely caused by infelicitous mapping to Metathesaurus concepts. For example, statin has two mapping candidates, “STN gene” and “Hydroxymethylglutaryl-CoA Reductase Inhibitors,” in the Metathesaurus. For most sentences, such as “… patients prescribed a statin with drugs that may increase the risk of myopathy”, “STN gene” was selected due to incorrect word sense disambiguation.

### Limitations and future work

Although our system can produce useful summaries for large numbers of MEDLINE citations and cluster the summary into several groups based on the themes, it has limitations. As mentioned in theme identification section, UCINET uses a hierarchical clustering algorithm to cluster cliques. Hierarchical clustering analysis is very practical in detecting topics for documents because it does not require human intervention to assign the number of the clusters in advance, as k-means clustering algorithm does. Wartena and colleagues [48] used a k-bisecting clustering algorithm, which is based on the k-mean algorithm, to cluster frequently occurring keywords in 758 documents taken from 8 Wikipedia categories. They clustered these into 9 categories, one for each Wikipedia category and one additional cluster. While in reality, it is almost impossible to pre-define the number of the clusters for varied topics in biomedical domain, Lee and colleagues [33] compared supervised and unsupervised methods to detect topics in biomedical texts and found that the performance of supervised topic spotting methods was better. They also found that unsupervised hierarchical clustering was robust and more readily applicable in real world settings. The clustering algorithm we used is based on the common concepts shared by the cliques. In other words, the clique-clique proximity matrix used for clustering is constructed on the basis of the similarity of predication arguments contained among the cliques. It ignores the similarity of predicates, which may also contribute to the computation of clique similarity. Although the effectiveness of clustering algorithms is not the focus of this paper, we will explore different clustering algorithms and consider adding predicates to enhance results in our future work.

**Figure 9:**
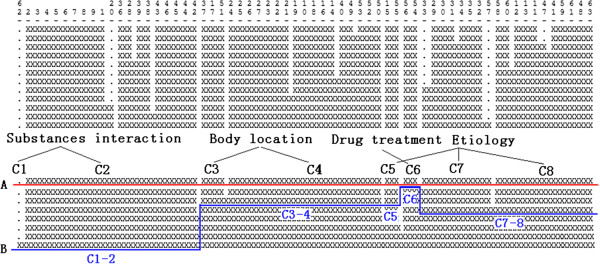
**Icicle plot for tumor necrosis factor.** The optimal cluster solution determined by our system is the row above the fixed threshold (line A). The theme label for each cluster for the system-determined solution is displayed. A dynamic cutoff B is displayed in blue.

Another limitation is that the final number of clusters is determined by a fixed threshold, which is widely used to detect the number of clusters in a dendrogram (cluster tree) whose branches are the clusters of interest. A fixed height on the dendrogram is chosen to cut the cluster tree into several groups. The icicle plot we used provides information similar to that in a dendrogram. We used semantic themes contained in each cluster to help find the height at which to cut the icicle plot. As mentioned in how to select the optimal clustering solution, when clusters identified in the icicle plot have different themes, the algorithm ends at that level and the corresponding row is determined to be the optimal clustering solution. But for some topics, the fixed threshold cannot achieve satisfactory results. For example, Figure 9 shows the icicle plot for clustering the topic tumor necrosis factor.

As shown in Figure 9, the system uses a fixed threshold (shown as line A) to group this topic into eight clusters. By considering the semantic information contained in each cluster, we can determine that the themes of cluster one and two are the same (substances interactions), cluster three and four are both about locations of the substances, while clusters five, seven and eight are all about chemicals as the cause of disorders; finally, cluster six is about chemicals treat disorders. It is obvious that repetitive themes are produced.

Instead of the fixed threshold, we will explore the use of a dynamic threshold [48] to detect clusters. Compared to cutoff based on fixed height, a dynamic threshold, which uses different cut heights on different branches of the cluster tree, makes determining the number of clusters more flexible. For example, [49] and [50] used a dynamic tree cut method on the basis of analyzing the shape of the branches of the dendrogram. In the future, we will consider both the shape of the icicle plot and cluster themes to determine a dynamic threshold, such as cutoff B in Figure 9. By considering the themes of clusters 1 to 8 in Figure 9, the dynamic cutoff B chooses different clustering solutions at different cutoff heights, so that clusters having the same cluster labels in the fixed threshold cutting method (clusters 1 and 2, clusters 3 and 4, and clusters 7 and 8) are merged together, and three new clusters (cluster1-2, cluster 3–4 and cluster7-8) are produced. With cutoff B, five clusters (marked in blue under the cutoff line) are produced for the topic TNF. Compared to cutoff A, dynamic cutoff B increases separation by 0.21 (0.52 versus 0.31) and overall validity by 0.14 (0.53 versus 0.39).

## Conclusion

We exploited graph theoretical methods to summarize biomedical documents; using hierarchical clustering, we then grouped the summary into several themes for a given topic based on the semantics contained in the summary. The system summary was compared to a reference standard produced by selecting the same number of the most frequent major MeSH descriptors as the number of concepts in the summary. The result showed that recall, precision and F-score were 0.64, 0.65 and 0.65 respectively. The validity of the clusters was compared to a baseline computed with the Silhouette Coefficient method for cohesion, separation and overall validity. The overall validity of the system output clusters was better than that of the Silhouette Coefficient clusters.

## Competing interests

The authors declare that they have no competing interests.

## Authors’ contributions

HZ conducted all research, including developing the algorithm and evaluation, and wrote the manuscript. MF provided suggestions to the research. DS implemented the algorithm. BW designed and implemented the algorithm for finding the quality of the cluster labeling (evaluation). TCR supervised the research and edited the manuscript. All authors read and approved the final manuscript.
